# An Unusual Cause of Acute Urinary Retention

**DOI:** 10.5811/cpcem.2018.2.37247

**Published:** 2018-04-05

**Authors:** Adam J. Smith, Paul Blackburn

**Affiliations:** Maricopa Integrated Health System, Maricopa Medical Center, Emergency Medicine Residency, Phoenix, Arizona

## CASE PRESENTATION

**Image 1:**
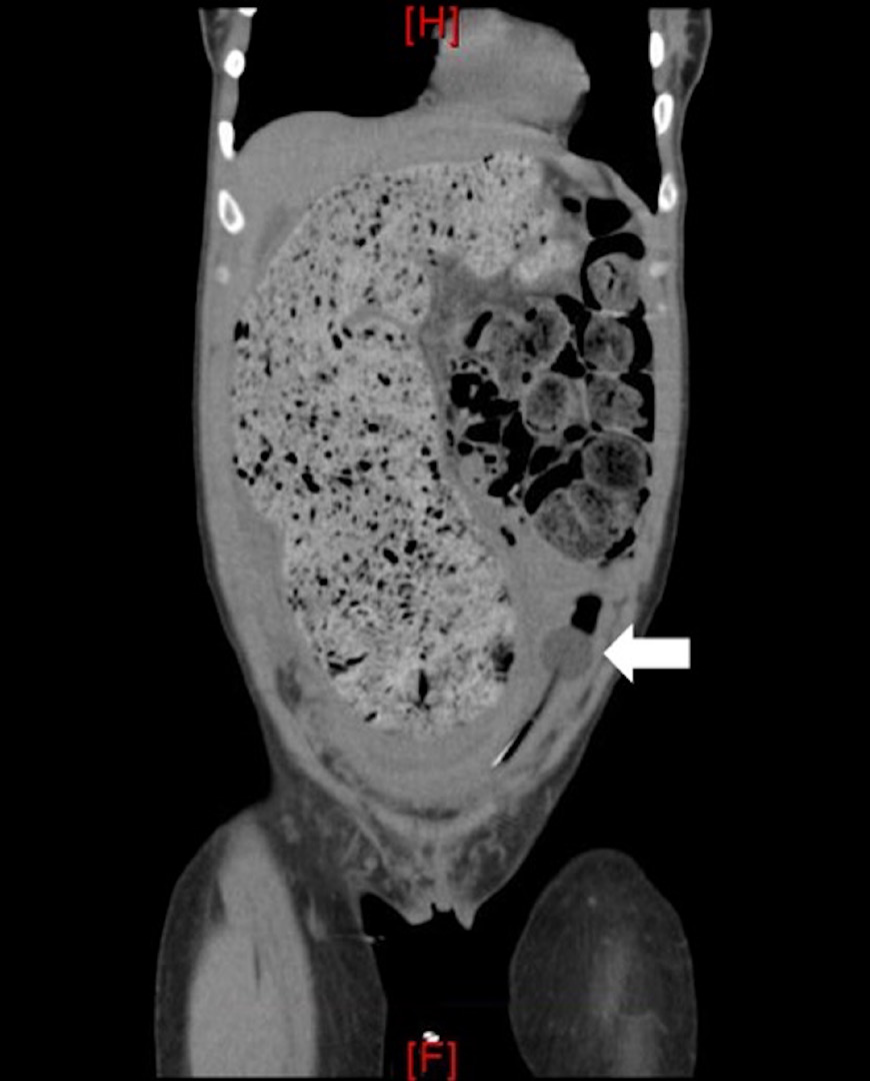
Computed tomography of the abdomen in the coronal plane demonstrating significant stool burden with displacement of the bladder to the left as noted by the urinary catheter bulb (arrow).

**Image 2:**
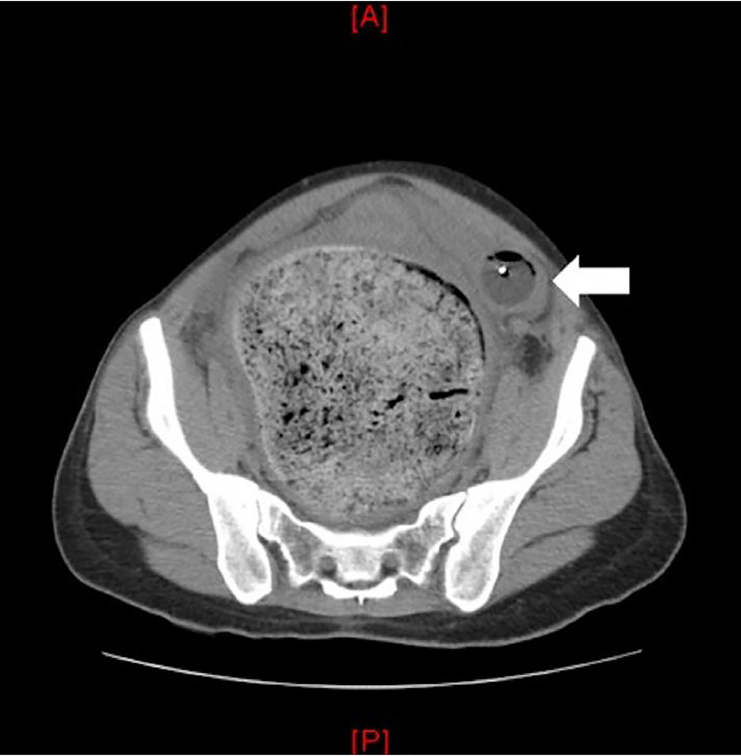
Computed tomography of the abdomen in the axial plane demonstrating stool burden causing significant displacement of the bladder as noted by the urinary catheter (arrow).

**Image 3:**
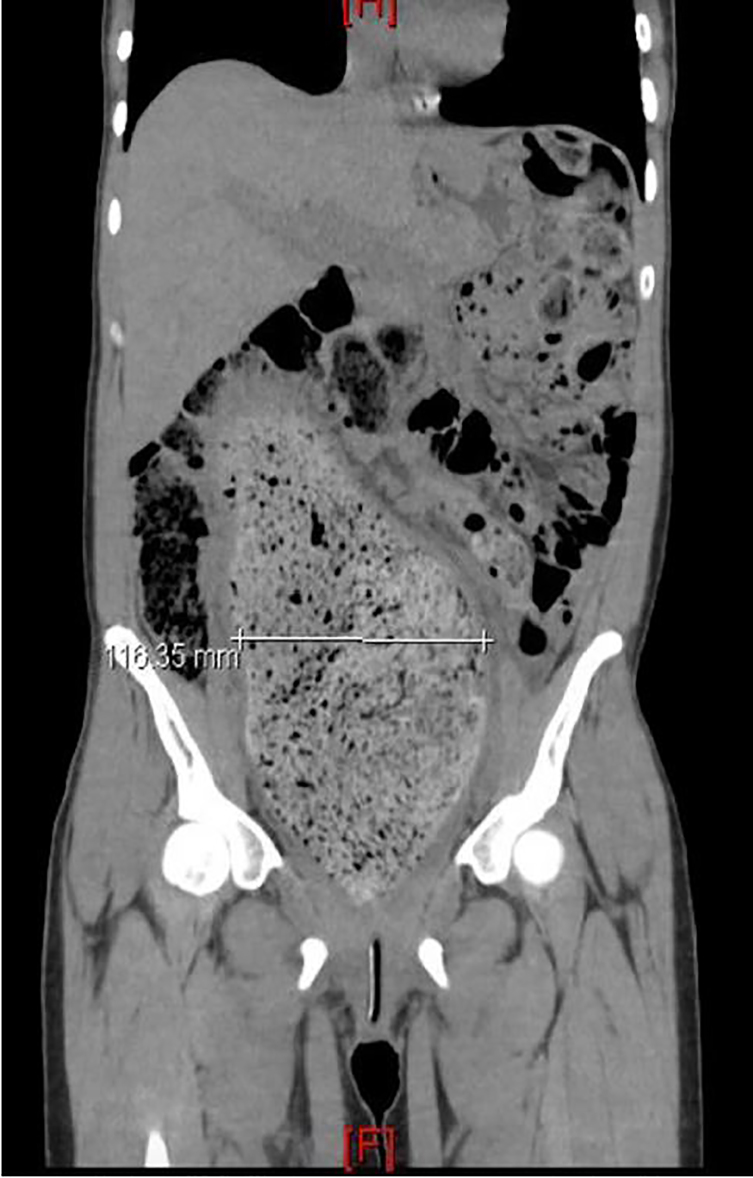
Computed tomography of the abdomen in the coronal plane demonstrating a colonic diameter of 116.35 mm at the level of the pelvic brim.

A 29-year-old female with a past medical history of constipation and anxiety, noted during previous pregnancies, presented with a chief complaint of acute urinary retention. She was not taking any medications and had no prior history of abdominal surgeries. She did report three previously uncomplicated pregnancies. Physical exam was significant for visible, firm suprapubic and right lower abdominal masses. Point-of-care ultrasonography demonstrated one liter of retained urine. An indwelling urinary catheter was inserted. The patient agreed to computed tomography (CT) for further evaluation (Images 1, 2, and 3).

## DISCUSSION

### Acute Urinary Retention Secondary to Chronic Constipation

CT imaging demonstrated severe idiopathic constipation causing megacolon and displacement of the bladder resulting in outlet obstruction. Acute urinary retention is uncommon in women with a prevalence of 1:100,000 women per year.^1^,^2^ Differential includes outflow obstruction, neurologic impairment, detrusor muscle weakness, medications (especially anticholinergics), and infection. Obstruction in women is generally secondary to anatomic distortion, including pelvic organ prolapse, pelvic masses, or urethral diverticulum.^1^,^3^

Constipation is an atypical cause of acute urinary retention in adults and is rarely mentioned in the literature.^4^ A sigmoid colon diameter of 6.5 cm at the pelvic brim is commonly used as a discriminating point for diagnosing megacolon.^5^ Treatment for severe chronic constipation and fecal impaction typically includes manual disimpaction and enemas, or oral solutions containing polyethylene glycol. Patients should receive Gastroenterology referral for colonic transit and motility studies. A patient may require partial colectomy if conservative medical therapy fails.^6^,^7^,^8^,^9^

**CPC-EM Capsule**

What do we already know about this clinical entity?

Acute urinary retention in women is most commonly secondary to anatomic distortion but may also be caused by medications, infection, and neurologic disease.

What is the major impact of the image(s)?

These images highlight the severity of the anatomic distortion that results from severe chronic constipation.

How might this improve emergency medicine practice?

Consider close follow up or admission for surgical and gastroenterology evaluation and to assess for other underlying causes and resolution of obstruction.

Documented patient informed consent and/or Institutional Review Board approval has been obtained and filed for publication of this case report.
